# Next-Generation Sequencing of the Complete Mitochondrial Genome of the Endangered Species Black Lion Tamarin *Leontopithecus chrysopygus* (Primates) and Mitogenomic Phylogeny Focusing on the Callitrichidae Family

**DOI:** 10.1534/g3.118.200153

**Published:** 2018-04-12

**Authors:** Patrícia Domingues de Freitas, Fernando Luis Mendez, Karla Chávez-Congrains, Pedro Manoel Galetti, Luiz Lehmann Coutinho, Alcides Pissinatti, Carlos Daniel Bustamante

**Affiliations:** * Departamento de Genética e Evolução, Universidade Federal de São Carlos, São Carlos, SP, Brazil; † Department of Genetics, Stanford University, Stanford, California, USA; ‡ Departamento de Zootecnia, EZALQ, Universidade de São Paulo, Piracicaba, São Paulo, Brazil; § Centro de Primatologia do Rio de Janeiro, Guapimirim, Rio de Janeiro, Brazil

**Keywords:** mtDNA, Phylogeny, Primates, Mitogenomics, Lion tamarins

## Abstract

We describe the complete mitochondrial genome sequence of the Black Lion Tamarin, an endangered primate species endemic to the Atlantic Rainforest of Brazil. We assembled the *Leontopithecus chrysopygus* mitogenome, through analysis of 523M base pairs (bp) of short reads produced by next-generation sequencing (NGS) on the Illumina Platform, and investigated the presence of nuclear mitochondrial pseudogenes and heteroplasmic sites. Additionally, we conducted phylogenetic analyses using all complete mitogenomes available for primates until June 2017. The single circular mitogenome of BLT showed organization and arrangement that are typical for other vertebrate species, with a total of 16618 bp, containing 13 protein-coding genes, 22 transfer RNA genes, 2 ribosomal RNA genes, and 1 non-coding region (D-loop region). Our full phylogenetic tree is based on the most comprehensive mitogenomic dataset for Callitrichidae species to date, adding new data for the *Leontopithecus* genus, and discussing previous studies performed on primates. Moreover, the mitochondrial genome reported here consists of a robust mitogenome with 3000X coverage, which certainly will be useful for further phylogenetic and evolutionary analyses of Callitrichidae and higher taxa.

The availability of high throughput DNA sequencing technology has prompted an increased interest in mitochondrial genomes. Aside from the understanding of their structural and gene organization, mitogenomes have been exploited for the reconstruction of robust phylogenies in different taxa (Zhang *et al.* 2003; Lin *et al.* 2015), including primates (Finstermeier *et al.* 2013; Malukiewicz *et al.* 2017).

The genus *Leontopithecus* contains four small primate species, which are allopatric and endemic to the Atlantic Forest of Brazil. These species are known as lion tamarins due to their prominent face-pelages, which resemble a lion’s mane (Rylands *et al.* 2002; Rylands and Anzenberger 2012). Studies of complete mitogenomes are almost nonexistent for this genus, with the exception of *Leontopithecus rosalia*, the Gold Lion Tamarin (GLT) (NC_021952) (Finstermeier *et al.* 2013). *Leontopithecus chrysopygus*, known as Black Lion Tamarin (BLT), exclusively inhabits the Atlantic Rainforest of São Paulo State (Rosenberger and Coimbra-Filho 1984), and is considered to be endangered by the International Union for Conservation of Nature (IUCN) (Kierulff *et al.* 2008).

Lion tamarins are members of the Callitrichidae Gray 1821, which includes other tamarins and also marmosets (Rosenberger and Coimbra-Filho 1984; Rylands and Mittermeier 2009). This family comprises around 60 species and subspecies from seven genera (*Callithrix* Erxleben 1777; *Saguinus* Hoffmannsegg 1807; *Mico* Lesson 1940; *Leontopithecus* Lesson 1840; *Cebuella* Gray 1866; *Callimico* Ribeiro,1922 and *Callibella* van Roosmalen 2003) that live in the tropical areas of Central and South America (Barroso *et al.* 1997; Rylands and Mittermeier 2009; Buckner *et al.* 2015; Rylands *et al.* 2016).

Several Callitrichidae species have been under threat mainly due to habitat fragmentation, gathering, and illegal trade. According to the Red List of IUCN, around 21 Neotropical primates from Platyrrhini parvorder are threatened by extinction. From this total, five species are considered the most endangered primates in the world (Schwitzer *et al.* 2014; IUCN 2017).

Despite numerous phylogenetic studies performed on platyrrhines in the last decades, issues involving evolutionary relationships among species and higher taxa remain unclear, and different phylogenies have been proposed for the Callitrichidae family (Barroso *et al.* 1997; Schneider 2000; Schneider *et al.* 2012; Harris *et al.* 2014). The commonly accepted consensus is that *Saguinus* and *Leontopithecus* were the first genera to separate from the others, and that *Callimico* and *Cebuella* are the closest sister groups of *Callithrix* (Kay 1990; Schneider *et al.* 1993, 2012; Schneider 2000; Opazo *et al.* 2006; Wildman *et al.* 2009; Springer *et al.* 2012; Duran *et al.* 2013; Finstermeier *et al.* 2013; Rosenberger *et al.* 2013; Harris *et al.* 2014). Some studies have also placed *Saguinus* as a sister group of *Leontopithecus* (Finstermeier *et al.* 2013). However, the phylogenetic reconstructions seem to be insufficiently resolved in this family.

Recent studies in primates have shown that mitochondrial-based phylogenies may provide more reliable information about evolutionary relationships among species and higher taxa than nuclear genes, and can also be successfully used to determine the timescale of their evolution (Finstermeier *et al.* 2013). Nevertheless, analyses of mitochondrial sequences (mtDNA) can reveal distinct results from nuclear sequences (nDNA) (Mundy and Kelly 2001; Perelman *et al.* 2011; Liedigk *et al.* 2014), although, both nDNA and mtDNA approaches have already shown congruent results (Schneider 2000; Schneider *et al.* 2012).

Alternatively, phylogenies using complete mitochondrial genomes may enable more robust statistical support when compared to analyses based on single genes (Liedigk *et al.* 2014). Recent studies using whole mitochondrial genomes of a wide range of primates have shown that mitogenomics may be more effective in defining some taxa species relationships than smaller mitochondrial fragment analyses (Malukiewicz *et al.* 2017). However, contrasting results have been found among representatives from the Callitrichidae family.

In this study, we characterized a high-coverage complete mitochondrial genome of *L. chrysopygus* and performed phylogenetic analysis using 124 complete mitogenomes available for the Primates order. The phylogenetic discussion focuses on the Callitrichidae family, from the Platyrrhini parvorder (Rylands and Mittermeier 2009); a full phylogeny has also been obtained for 65 Old World Monkeys (OWMs) and 34 Strepsirrhini, providing a highly robust mitogenomic phylogeny for Primates, and adding new data for callitrichids. Our complete tree includes 90 Haplorrhini species. Of these, 25 are New World Monkeys (NWMs) and nine are from the Callitrichidae family.

## Material and Methods

### Ethical statement

Sample collection followed all ethical requirements proposed by the American Society of Primatologist for the Ethical Treatment of Non-Human Primates, and was approved by SISBIO #50616-1 (Authorization System and Biodiversity Information, Chico Mendes Institute for Biodiversity Conservation, Ministry of Environment, Brazil), and CEUA #9805200815 (Ethics Committee on Animal Experimentation and Research, UFSCar, São Carlos, São Paulo, Brazil).

### Sample collection

The biological sample of *L. chrysopygus* was obtained from the Primatology Center of Rio de Janeiro (CPRJ), located in Guapimirim (Rio de Janeiro, Brazil). One adult male Black Lion Tamarin, who was born in captivity in 2007, was anesthetized using an inhalation mask with Isoflurane (2%) and Oxygen (2 L/minute), and then, 2 mL of peripheral venous blood were collected with *vacutainer* containing EDTA (3.6 mg). The sample was stored at -20°, and then used for DNA extraction.

### DNA extraction, next generation sequencing experiments

Total Genomic DNA was extracted using a ReliaPrep Blood gDNA Miniprep System Kit (Promega, Fitchburg, WI, USA), and DNA quality and quantity were evaluated on a NanoDrop Spectrophotometer (Thermo Fischer, Waltham MA, US). About 2 ug of DNA were used to construct short-insert libraries using a Nextera DNA Library Prep Kit (Illumina, San Diego, CA, USA). A HiSeq SBS Kit v4 PE was used to sequence runs of paired-end reads (2 × 101 bp) on the HiSequation 2500 Illumina Platform (Illumina, San Diego, CA, USA).

### Mitogenome assembly

The mitogenome of the BLT was assembled in several steps. First, we employed the LeeHom tool (Renaud *et al.* 2014) to merge read pairs with overlapping of ten or more bases. Then, we trimmed low-quality bases using an in-house program. Trimmed sequences were preserved only if they were at least 30 bases long. We mapped the reads using mtDNA sequences of four close relatives of BLT (*Callithrix jacchus*; *Callithrix pygmaea*, here named as *Cebuella*; *Saguinus oedipus*; and *Leontopithecus rosalia*) as references (see Table S1) and the assembler Velvet 1.2.10 (Zerbino and Birney 2008). To obtain a contiguous sequence, we implemented the De Brujin graph approach, using long k-mers. More details for the complete mitogenome assembly are described in the Supplemental Material (see Appendix S1).

### NUMTs and heteroplasmies searching

We mapped the fastq reads to our consensus sequence and generated pileup files using SAMtools (Li *et al.* 2009; Li 2011). For almost 600 positions, the consensus base was seen less than 99% of the time. We then filtered out each read (and its partner if it was a read pair) with edit distance of two or more from the consensus sequence. After this filtering step, we only observed three positions in which the consensus was seen less than 99% of the time. We then filtered out each read (and its partner if it was a read pair) with soft-clipped bases. These bases are typical of the boundary between a NUMT (Nuclear Mitochondrial pseudogene) and the rest of the chromosomal sequence. After filtering these reads, and considering alternate bases seen at least three times, we observed the most frequent alternative base was present at a frequency of less than 0.25% (supported by 7 reads maximum), allowing us to assess the presence of heteroplasmies (see Appendix S1).

### Mitogenome characterization and phylogenetic analyses

We performed an initial automatic annotation in the MITOS webserver (Bernt *et al.* 2013). Next, we conducted a more accurate annotation in the Bioedit software (Hall 1999, 2011), using both *L. rosalia* (NC_021952) and *Homo sapiens* (NC_012920) mitochondrial genomes as references.

We used tRNAscan-SE Search Server v.1.21 (Lowe and Eddy 1997) and foldRNA webserver (Gruber *et al.* 2008) to predict tRNA secondary structures. We calculated the mitogenome nucleotide composition, and AT and GC skews using CountBasesDNA (https://github.com/KChavez-Congrains/CountBasesDNA). The illustration for the complete mitogenome was performed on the BRIG program (Alikhan *et al.* 2011).

We downloaded all complete mitogenomes available for 123 primate species, collected by June 2017, and also of three other mammal species using the NCBI’s taxonomy browser (https://www.ncbi.nlm.nih.gov/genome) (see Table S1) and aligned them using the MAFFT program (Katoh and Standley 2013). We used the parameter Translator X (Abascal *et al.* 2010) for protein coding regions and concatenated them. We conducted Maximum Likelihood (ML) analyses using RAxML (Stamatakis 2014) and implemented the GTR+G model for the partition identified in the Partition Finder (Lanfear *et al.* 2012).

### Data availability

The complete mitochondrial genome sequence described here is available at GenBank (https://www.ncbi.nlm.nih.gov/genbank/) under accession number MG933868. Supplemental Material was uploaded on Figshare data repository (https://figshare.com/s/5d20d00529afaf60f390). Figure S1 contains the quartiles for the distribution of fragment lengths for the mitochondrial genome assembly; Figure S2 contains the llustration for the tRNA mitochondrial; Figure S3 contains the full phylogenetic tree, using Maximum Likelihood for 124 primates and three outgroup species; Table S1 contains the list of the GenBank accession numbers for the complete mitochondrial genome sequences previously published by different authors and used in our phylogenetic analysis. Details of the methodology employed to perform the mitogenome assembly, and NUMTs and heteroplasmy searching are described in Appendix S1. Supplemental material available at Figshare: https://figshare.com/s/5d20d00529afaf60f390.

## Results and Discussion

### Mitogenome organization and nucleotide composition

**Figure 1:**
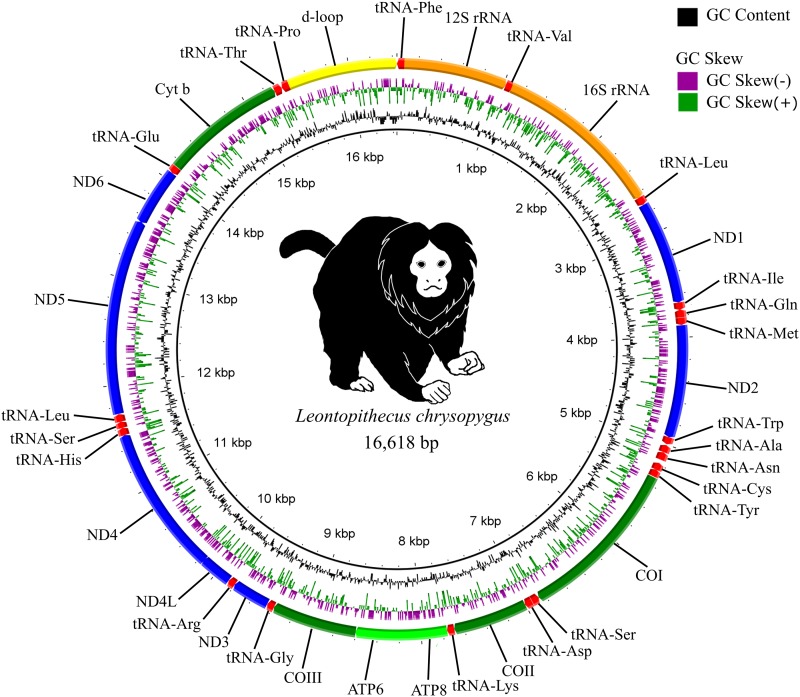
Illustration for the complete mitochondrial genome of the Black Tamarin *Leontopithecus chrysopygus*, showing the relative position of the mitochondrial sequences and GC skew and content.

The mitogenome of *L. chrysopygus* was assembled, with ∼3000X coverage, as a single circular molecule of 16618 bp (Figure 1), which is comparable to other Callitrichidae mitochondrial genomes, including the 16,499 bp of *C. jacchus* and 16,872 bp of *L. rosalia* (Finstermeier *et al.* 2013; Malukiewicz *et al.* 2017). We do not report any heteroplasmy after the several filtering steps (see Figure S1). We annotated 37 genes, including 13 protein coding, 22 tRNA, and 2 rRNA genes (Table 1).

**Table 1:** Description of the mitochondrial genes for *Leontopithecus chrysopygus,* including information related to gene size and position (bp), strand-location (heavy/light), and their respective start and stop codons and anticodons

Gene	Position		Size (bp)	Strand	Codon		Anticodon
	From	To			Start	Stop	
tRNA-Phe	1	69	69	Heavy			GAA
12S-rRNA	70	1022	953	Heavy			
tRNA-Val	1023	1090	68	Heavy			TAC
16S-rRNA	1081	2646	1556	Heavy			
tRNA-Leu	2647	2721	75	Heavy			TAA
ND1	2724	3679	956	Heavy	ATG	TA	
tRNA-Ile	3680	3748	69	Heavy			GAT
tRNA-Gln	3817	3746	72	Light			TTG
tRNA-Met	3821	3888	68	Heavy			CAT
ND2	3891	4929	1039	Heavy	ATT	T	
tRNA-Trp	4930	4996	67	Heavy			TCA
tRNA-Ala	5074	5006	69	Light			TGC
tRNA-Asn	5148	5076	73	Light			GTT
tRNA-Cys	5248	5182	67	Light			GCA
tRNA-Tyr	5314	5248	67	Light			GTA
COI	5322	6863	1542	Heavy	ATG	TAG	
tRNA-Ser	6933	6865	69	Light			TGA
tRNA-Asp	6937	7005	69	Heavy			GTC
COII	7006	7693	688	Heavy	ATA	T	
tRNA-Lys	7694	7760	67	Heavy			TTT
ATP8	7761	7967	207	Heavy	ATG	TAG	
ATP6	7922	8602	681	Heavy	ATG	TAA	
COIII	8602	9385	784	Heavy	ATG	T	
tRNA-Gly	9386	9452	67	Heavy			TCC
ND3	9455	9800	346	Heavy	ATA	T	
tRNA-Arg	9801	9866	66	Heavy			TCG
ND4L	9869	10165	297	Heavy	ATG	TAA	
ND4	10159	11533	1375	Heavy	ATG	T	
tRNA-His	11534	11602	69	Heavy			GTG
tRNA-Ser	11603	11661	59	Heavy			GCT
tRNA-Leu	11662	11732	71	Heavy			TAG
ND5	11736	13541	1806	Heavy	ATA	TAA	
ND6	14071	13538	525	Light	ATG	TAA	
tRNA-Glu	14140	14072	69	Light			TTC
Cyt b	14145	15284	1140	Heavy	ATG	TAA	
tRNA-Thr	15286	15355	70	Heavy			TGT
tRNA-Pro	15426	15358	69	Light			TGG
D-loop	15427	16618	1192				

The heavy strand contains 12 protein-coding genes, 14 tRNAs and 2 rRNAs, whereas the light strand contains one protein-coding gene and 8 tRNAs. The gene order found herein follows the typical arrangement described for other primates (Malukiewicz *et al.* 2017). The 13 protein-coding genes present three different start codons: ATG (ND1, COI, ATP8, ATP6, COIII, ND4L, ND4, ND6, and Cytb), ATA (COII, ND3, ND5), and ATT (ND2); and seven genes show the complete termination codons: TAG (COI, ATP8), and TAA (ATP6, ND4L, ND5, ND6, Cytb). There are twenty-two tRNAs coding genes in the mitogenome of BLT. The tRNASer (GCT) did not exhibit the typical clover-leaf structure, containing the dihydrouridine (DHU) arm (see Figure S2).

The D-loop region presents a total length of 1192 bp, and contains an STR region (TA)_14_ between the nucleotides 957 and 984. Seventeen inter-genic spacers were found to have a total length of 75 bp, ranging from 1 to 35 bp, with the longest located between tRNA-Asn and tRNA-Cys. Gene-overlap is observed between fifteen contiguous genes, by a total of 76 bases ranging from 1 to 46 bp. Overlapping genes for tRNA-Ile and tRNA-Gln and for ND5 and ND6 are encoded in opposite strands.

The composition of the *L. chrysopygus* mtDNA is biased toward adenine and thymine. The proportion of A+T content is 62.65% for protein-coding genes, 65.64% for tRNAs, 60.54% for rRNAs, and 65.52% for the D-loop region. The protein-coding genes have almost equal amounts of A and T; however, they are GC-skewed. The A+T content increases and the GC-skew decreases with codon position. tRNAs preferably contain A and G, while rRNAs have a greater fraction of A and C (Table 2).

**Table 2:** Information for the complete mitochondrial sequence of *Leontopithecus chrysopygus*, describing the proportion of nucleotides (percentage of A, T, C and G) and Skew values of AT and GC

		Proportion of nucleotides						Skew	
		%A	%T	%G	%C	%A+T	%G+C	AT	GC
Whole mitogenome		34.30	26.65	15.53	23.52	60.95	39.05	0.13	−0.20
Protein-coding genes		31.31	31.34	12.25	25.10	62.65	37.35	0.00	−0.34
	First codon position	31.72	24.25	21.32	22.71	55.97	44.03	0.13	−0.03
	Second codon position	19.87	41.13	10.99	28.01	61.00	39.00	−0.35	−0.44
	Third codon position	42.36	28.63	4.41	24.60	70.99	29.01	0.19	−0.70
Protein-coding genes-H strand		31.29	31.34	12.24	25.13	62.63	37.37	0.00	−0.34
	First codon position	31.72	24.25	21.32	22.71	55.97	44.03	0.13	−0.03
	Second codon position	19.87	41.13	10.99	28.01	61.00	39.00	−0.35	−0.44
	Third codon position	42.36	28.64	4.41	24.59	71.00	29.00	0.19	−0.70
Protein-coding genes-L strand		23.22	40.64	26.40	9.74	63.86	36.14	−0.27	0.46
	First codon position	27.53	26.40	38.20	7.87	53.93	46.07	0.02	0.66
	Second codon position	16.85	43.26	20.79	19.10	60.11	39.89	−0.44	0.04
	Third codon position	25.28	52.25	20.22	2.25	77.53	22.47	−0.35	0.80
tRNA genes		33.93	31.61	18.62	15.84	65.54	34.46	0.04	0.08
	tRNA genes-H strand	36.79	30.51	16.14	16.56	67.30	32.70	0.09	−0.01
	tRNA genes-L strand	29.01	33.51	22.88	14.60	62.52	37.48	−0.07	0.22
rRNA genes		35.25	25.29	17.63	21.83	60.54	39.46	0.16	−0.11
D-loop		31.29	34.23	12.25	22.23	65.52	34.48	−0.04	−0.29

Although characterization of mitochondrial genomes has sustainedly increased in recent years, the number of complete mitogenomes for primate species is minuscule in light of the extreme importance and high species diversity of this group. This is especially true when we consider the remarkable utility of mitochondrial data in resolving phylogenetic relationships among taxa, including those with recent divergence time, and detecting evolutionary events involving gene duplication, loss, and rearrangements. Comparative analyses of complete mitogenomes can also be successfully used to provide insights into adaptive processes (Finstermeier *et al.* 2013; Oceguera-Figueroa *et al.* 2016; Wang *et al.* 2016). Despite this, there are currently only 65 complete mitochondrial genomes described for catarrhines, and 24 for platyrrhines. Of those, eight are from the Callitrichidae family and one from the genus *Leontopithecus*.

### Phylogenetics

Our phylogenetic analysis (ML) included 127 whole mitochondrial genomes, comprising 124 primates from 16 families and tree outgroup species. The full phylogenetic tree showed the two largest typical primate clades, separating Haplorrhini (composed by Simiiformes and Tassiformes) from Strepsirrhini (Lorisiformes, Chiromyiformes and Lemuriformes) (Finstermeier *et al.* 2013). Among the Simiiformes, Platyrrhini was separated from Catarrhini parvorder, as expected (see Figure S3).

New World monkeys from Platyrrhini parvorder are differentiated from Old World monkeys and apes from Catarrhini parvorder, especially due to the remarkable presence of prehensile tails and flat noses (Hershkovitz 1977; Ford 1980; Rosenberger *et al.* 2013). Unlike other platyrrhines, marmosets (*Callithrix*, *Mico*, *Cebuella*, *Calibella*), tamarins (*Saguinus*, *Leontopithecus*) and callimicos (*Callimico*) from the Callitrichidae family have only big toes with sharp nails. The other fingers have claws (clawed members) that are used for arboreal activities (Ford 1980; Rylands and Mittermeier 2009; Rosenberger *et al.* 2013). Moreover, callitrichids have diminutive corporeal sizes and propensity to produce twins and triplets, with the exception of *Callimico* (Goeldi’s monkey) that produces only single births, and that did not lose the third molar (Hershkovitz 1977; Harris *et al.* 2014). Despite this, taxonomic studies using morphological, reproductive, and molecular data have indicated that *Callimico* is a sister group of marmosets (Barroso *et al.* 1997; Harris *et al.* 2014; Buckner *et al.* 2015; Schneider and Sampaio 2015).

**Figure 2:**
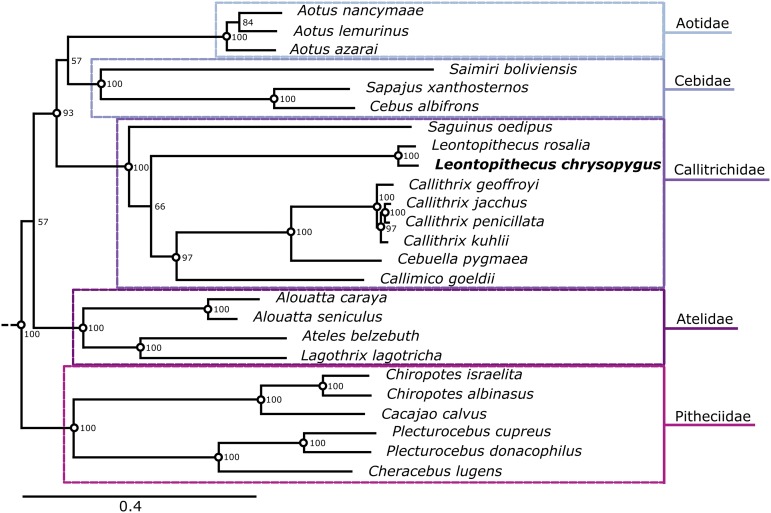
Phylogenetic tree based on Maximum Likelihood obtained for Platyrrhini parvorder, using complete mitochondrial genomes of 25 species from Aotidae, Cebidae, Callitrichidae, Atelidae, and Pitheciidae family.

In regards to the Calltrichidae family, our phylogenetic tree links *Callithrix* to *Cebuella* and places *Callimico* as the sister group to *Callithrix/Cebuella*, supported by high bootstrap values (100 and 97, respectively). *Saguinus* appears as the basal genus among the callitrichines, as is the case in other studies previously performed in Callitrichidae (Schneider 2000; Opazo *et al.* 2006; Wildman *et al.* 2009; Springer *et al.* 2012; Menezes *et al.* 2013; Rylands *et al.* 2016; Malukiewicz *et al.* 2017). However, our data did not support the characterization of *Saguinus* and *Leontopithecus* as sister groups (Figure 2).

Previous studies using nDNA and mtDNA sequences have described *Saguinus* and *Leontopithecus* as non-sister groups. Wildman *et al.* (2009) obtained a well-supported tree with non-coding genomic regions, which exhibits the same relationship found by Opazo *et al.* (2006), using seven nuclear genes. Springer *et al.* (2012) reported similar results for *Saguinus*, *Leontopithecus*, *Callimico*, and *Callithrix* when they performed a concatenated phylogenetic analysis using both mtDNA and nDNA data. In a recent study based on nuclear data produced by Perelman *et al.* (2011), Rylands *et al.* (2016) also report *Saguinus* as a non-sister group of *Leontopithecus*, and *Callithrix* as more closely related to *Cebuella*, as we also found in this study. *Callimico* and *Mico* form a clade that is linked to that of *Callithrix*/*Cebuella*. Although we had not included *Mico* in our phylogenetic analysis, due to the absence of a complete mitogenome in this genus, our tree is more congruent with these arrangements than with the complete mitogenome phylogenetic analyses performed by Finstermeier *et al.* (2013) and Malukiewicz *et al.* (2017), which place *Leontopithecus* as a sister group of *Saguinus*.

In sum, our phylogenetic tree of the Callitrichidae family results in a well-supported monophyletic group. Nonetheless, the internal phylogeny does not support the *Leontopithecus* genera as a sister group of *Saguinus*.

### Conclusions

In this study, we have successfully assembled (with high coverage) the whole mitochondrial genome of *L. chrysopygus*, and have obtained a well-resolved phylogeny for primates based on all the protein-coding mitochondrial genes. These data decisively contribute to our knowledge of the evolutionary relationships within Callitrichidae and can be useful in further understanding the phylogenetic and evolutionary relationships within Callitrichidae and higher taxa. Considering that *Leontopithecus* is a rare endangered genus, understanding its phylogenetic relationships within Callitrichidae can also be beneficial to the conservation of these animals in cases where management decisions depend upon robust phylogeny.
